# Rational design of anti-Kasha photoemission from a biazulene core embedded in an antiaromatic/aromatic hybrid†

**DOI:** 10.1039/d3sc00405h

**Published:** 2023-05-22

**Authors:** Aitor Diaz-Andres, Jose Marín-Beloqui, Junting Wang, Junzhi Liu, Juan Casado, David Casanova

**Affiliations:** a Donostia International Physics Center (DIPC) 20018 Donostia Euskadi Spain david.casanova@dipc.org; b Department of Chemistry, State Key Laboratory of Synthetic Chemistry, The University of Hong Kong Pokfulam Road Hong Kong China; c Department of Physical Chemistry, University of Malaga Campus de Teatinos s/n 29071 Malaga Spain; d IKERBASQUE – Basque Foundation for Science 48009 Bilbao Euskadi Spain

## Abstract

The violation of the Kasha photoemission rule in organic molecules has intrigued chemists since their discovery, being always of relevance given its connection with unique electronic properties of molecules. However, an understanding of the molecular structure–anti-Kasha property relationship in organic materials has not been well-established, possibly because of the few existing cases available, limiting their prospective exploration and *ad hoc* design. Here we introduce a novel strategy to design organic emitters from high excited states combining intramolecular *J*-coupling of anti-Kasha chromophores with the hindering of vibrationally-induced non-radiative decay channels by enforcing molecular rigidity. We apply our approach to the integration of two antiparallel azulene units bridged with one heptalene all inserted into a polycyclic conjugated hydrocarbon (PCH). With the help of quantum chemistry calculations, we identify a suitable PCH embedding structure and predict its anti-Kasha emission from the third high energy excited singlet state. Finally, steady fluorescence and transient absorption spectroscopy studies corroborate the photophysical properties in a recently synthesized chemical derivative with this pre-designed structure.

## Abstract 2

Design of anti-Kasha emitters based on the *J*-coupling of azulenes and molecular rigidity imposed by antiaromatic/aromatic embedding.

## Graphical abstract

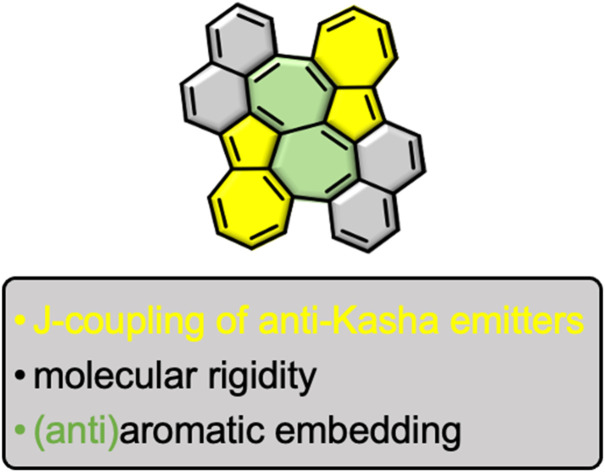

## Introduction

Light emission (fluorescence or phosphorescence) in organic molecules, in the vast majority of cases, proceeds from the lowest energy excited state irrespective of the excitation energy used.^1–3^ This is a consequence of the energy gap law based on the fact that closely packed excited states, that is, with small energy separations, always lie with strong vibronic overlaps promoting efficient S_*n*_ → S_1_ non-radiative processes, *i.e.*, internal conversion (IC). Contrarily, when the interstate gaps are large, radiative transitions can emerge as the most efficient relaxation mechanisms, as it is usually the case for the S_1_ → S_0_ decay. This is known as the Kasha's rule, which states that most of the molecules are emissive from the lowest energy, same (ground state) spin multiplicity, S_1_ excited state.^4^ Since it was claimed by Michael Kasha in 1950, few cases with emission from higher excited states have appeared, also known as anti-Kasha emitters.^5–10^ Azulene, the non-alternant isomer of naphthalene, and its derivatives form a paradigmatic family of anti-Kasha emitters.^5,8–14^ Azulene exhibits fluorescent emission from the second excited singlet state (S_2_), mainly triggered by: (i) the large energy separation with S_1_ (preventing efficient S_2_ → S_1_ IC), and (ii) the larger oscillator strength of S_2_ (S_2_ → S_0_) compared to S_1_ (S_1_ → S_0_). These two factors allow S_2_-fluorescence to compete with IC.

A potential strategy to expand the pool of molecules endowed with anti-Kasha luminescence is *via* integration of azulene-like fragments in polycyclic conjugated hydrocarbons (PCHs). PCHs with non-alternant and antiaromatic topologies have constantly attracted the curiosity of chemists^15–17^ owing to their unique electronic,^18–20^ optical^21,22^ and structural features^23–28^ in comparison with their respective polycyclic aromatic/benzenoid hydrocarbons (PAHs).

Recently, non-alternant PCHs have engaged renewed interest due to their role as chemical defects,^29^*i.e.*, Stone–Wales, in nanographene topological insulators and semiconductors,^30,31^ graphene quantum dots,^32,33^ or as spin qubits.^30^ Modular bottom-up organic synthesis has constructed several PCHs containing one or multiple azulene units,^34^ showing exotic photophysical and electronic properties.^19,22,23,25,26,35–40^ However, most of the known PCHs with azulene fragments in expanded forms do not show the intrinsic azulene-like anti-Kasha fluorescence, since, among other factors, benzenoid rings surrounding the azulene core and included for thermodynamic stabilization increase the local aromatic character what adversely bleaches anti-Kasha conditions.^38,41^ The task to encounter molecules with a fine balance between aromatic/non-alternant composition able to be stable and simultaneously conserve the anti-Kasha anomaly is certainly challenging. A comprehensive understanding of the modes of manipulation of light absorption and emission over broader wavelength ranges of the electromagnetic spectrum, to which anti-Kasha emitters can contribute, is a key aspect for the design of next-generations organic fluorophores able to serve as more versatile and efficient energy conversion substrates.

**Fig. 1:**
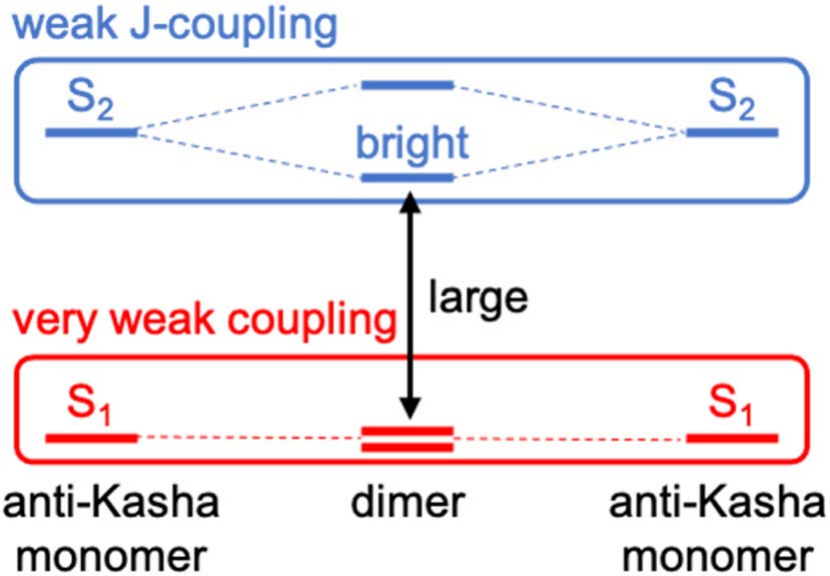
Molecular design strategy based on the coupling of non-Kasha emitters with weak *J*-coupling between high-energy (anti-Kasha) states (in blue) and very weak interaction between lowest excited states (in red).

To design new molecules with anti-Kasha fluorescence, two aspects should be considered: (i) to selectively increase the optical activity of a given high-energy singlet excited state from which fluorescence will detune; and (ii) to promote the conditions for inefficient IC towards lowest-lying excited singlets. For this twofold purpose, we herein conceive a design strategy for a bright S_3_ state based on the coupling of two equivalent non-Kasha emitters, *e.g.*, azulene. Firstly, to enhance the optical activity of the high-energy excited state, we borrow the *J*-coupling concept from supramolecular photophysics, in which “head-to-tail” stacked dimers induce an augment of radiative decay rates (larger oscillator strengths).^42^ In this sense, we apply the Kasha's excitonic coupling model,^43,44^ based on the interaction of transition dipole moments, to the coupling of the S_2_ state of azulene in order to design new dimeric azulene compounds with the suitable disposition between the two S_2_ transition dipole moments (Fig. 1). Secondly, to hinder IC from the bright state to lower spin-singlet states, we intend to preserve a large energy gap with respect to same-spin states. For that, it is desirable to have a (*J*-like) weak coupling of the two local S_2_ transitions, and simultaneously a vanishing interaction between S_1_ states of the building monomers. Finally, we explore the structural embedding of the target dimer in various PCHs in order to ensure antiparallel planar disposition of the two azulenes and enforce structural rigidity to further hinder non-radiative decay pathways.

Our study relies on the results from quantum chemical calculations of potentially suitable dimeric azulene structures, *i.e.*, biazulenes. In this article, we find out that a *J*-type parallel coupling of two azulene units through the lateral positions of five member rings gathers the conditions for anti-Kasha emission from a bright S_3_ excited state. Moreover, we identify a proper PCH embedding for the optimal biazulene core that preserves the requirements for high-excited state emission. Finally, the predicted photophysical properties are experimentally corroborated in a recently synthesized derivative of the computationally designed anti-Kasha emitter.

## Experimental details

### Electronic structure calculations

Hückel molecular orbital energies were obtained using the orbital parameters *α* (the energy of an electron in a p_*z*_-orbital) and *β* (the interaction energy between two vicinal orbitals) from the reported literature values.^45^ Ground-state molecular geometries have been optimized at the density functional theory (DFT) level with the CAM-B3LYP functional^46^ and the cc-pVDZ basis set. Vertical electronic transitions and excited state minima were obtained with the time-dependent version of DFT (TDDFT), by using a larger basis set (cc-pVTZ) and with the rCAM-B3LYP functional, which has shown to minimize many-electron self-interaction error in largely conjugated systems.^47^ Nucleus-independent chemical shift (NICS)^48^ calculations have been done at the same computational level. All calculations have been performed with the Q-Chem package.^49^

### Fluorescence and transient absorption measurements

Femtosecond transient absorption spectroscopy characterization was carried out with a Helios equipment from Ultrafast Systems, equipped with an amplified femtosecond Spectra-Physics Solstice-100F laser (with a 128 fs pulse width and 1 kHz repetition rate) coupled with a Spectra-Physics TOPAS Prime F optical parametric amplifier (195–22 000 nm). Samples were studied in CH_2_Cl_2_ solutions. The excitation power was modified using neutral density filters to match the desired power.

## Results and discussion

### Optimal covalent arrangement for S_3_ anti-Kasha emission

**Fig. 2:**
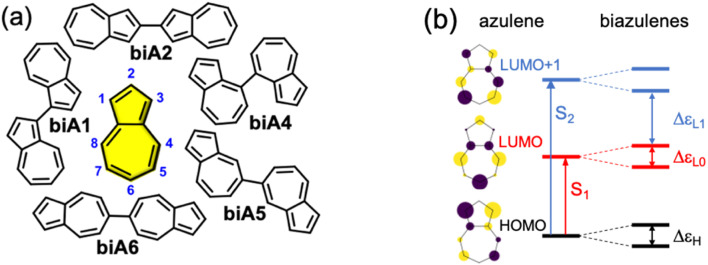
(a) Molecular structures of azulene (yellow) and symmetrical biazulenes (biA1–biA6). (b) Hückel-type molecular orbital diagram for azulene and biazulenes. Vertical arrows indicate orbital transitions for S_1_ and S_2_ states in azulene. Double arrows indicate orbital gaps in biazulenes.

Firstly, we explore possible linking modes in covalent dimers of azulene in order to generate the proper interstate couplings and a state energy diagram similar to the one envisaged in Fig. 1. For that, we devise five symmetrically connected dimers, biA1–biA6 (Fig. 2a). Interestingly, the optoelectronic and charge-transport properties of some of these symmetric biazulenes (biA1 and biA6) have been recently characterized.^50^ Asymmetric biazulene arrangements, *i.e.*, connected by different azulene positions, are disregarded, since they should be prone to induce exciton localization, recovering (at most) the emission properties of the monomer. In other words, the bright excited state (S_3_) should delocalize over the two non-Kasha moieties, which require the two monomers to be equivalent. Therefore, only symmetrical dimers, *i.e.*, connected by the same azulene positions, are considered. Besides, we are interested in orientations of the two local transition dipole moments of S_2_ of azulene (aligned along the long molecular axis) resulting in *J*-type interactions, so the *syn*-forms of biazulenes, expected to trigger *H*-coupling, are not taken into account here.

To achieve a bright S_3_ excited state, we aim to establish simple guidelines for biazulene violating Kasha's rule based on the magnitude of orbital couplings. The Hückel molecular orbitals of azulene and the general orbital distribution in biazulenes are presented in Fig. 2b. The two lowest excited singlet states of azulene correspond to the HOMO-to-LUMO (S_1_) and HOMO-to-LUMO+1 (S_2_) electronic promotions (Fig. 2b). Within the one-electron picture, we can associate the S_2_/S_3_ gap in biazulene with the energy difference between LUMO+1 and LUMO+2 (Δ*ε*_L1_ in Fig. 2b). Strong interaction between the two LUMOs of azulene would result in large destabilization of the LUMO+1 of the dimer, which will shrink Δ*ε*_L1_ and accelerate IC decay from S_3_. Hence, a small LUMO/LUMO+1 gap in the dimer (Δ*ε*_L0_) is desirable, which requires a weak coupling between LUMOs of each azulene. On the other hand, degeneracy between the two highest occupied orbitals (Δ*ε*_H_ = 0) might induce an increase of the density of states, *e.g.*, low-energy HOMO−1 → LUMO transition, favoring non-radiative processes. Additionally, to avoid orbital mixing with the π-system of the peripheral conjugated rings when embedded into a PCH (see below), a high energy HOMO on the biazulene is desired, which requires a large Δ*ε*_H_, *i.e.*, a strong coupling between HOMOs of each azulene. Therefore, considering all these conditions, we predict that anti-Kasha emission from S_3_ will be facilitated in those dimers with: large Δ*ε*_L1_ and Δ*ε*_H_, and small Δ*ε*_L0_.

**Fig. 3:**
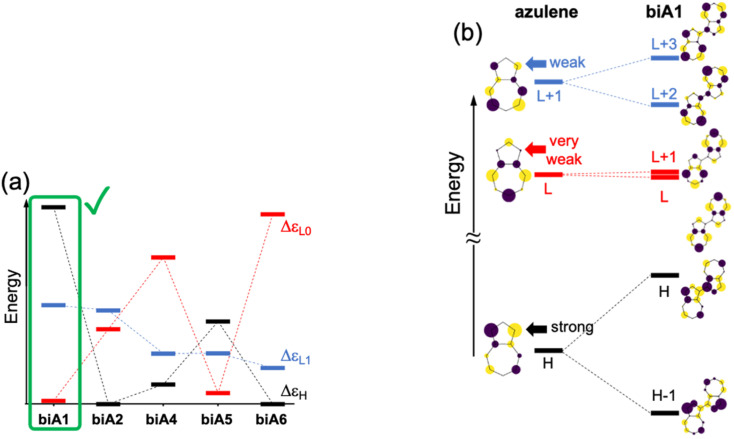
(a) Orbital energy differences (Δ*ε*_H_, Δ*ε*_H_, and Δ*ε*_L1_) for symmetrical biazulenes (biA1–biA6). (b) Molecular orbital diagrams of azulene and biA1. Participation of p_*z*_-orbital in position 1 of azulene highlighted. Energy gaps and orbitals obtained with the Hückel model. H = HOMO; L = LUMO.

The molecular orbital energy differences for the five symmetric biazulenes are presented in Fig. 3a as obtained by the Hückel model. The biA1 dimer (antiparallel azulenes linked at the 1,1-position) presents the largest Δ*ε*_L1_ and Δ*ε*_H_, and a tiny Δ*ε*_L0_, hence, according to the requirements discussed above, biA1 is identified as the most suitable arrangement for (S_3_) non-Kasha emission. These results can be rationally understood by the π-electron density at the connecting positions, which are optimal in position 1 (Fig. 3b): large in the HOMO (strong coupling), vanishing in the LUMO (no interaction), and small in LUMO+1 (weak coupling). It is also important to notice that the 1,1-linkage in biA1 triggers antibonding (bonding) interaction in the HOMO (LUMO+2) and ensures that the HOMO → LUMO+2 excitation corresponds indeed to a “head-to-tail” (like) orientation of the transition dipole moments on each azulene, predicting a bright S_3_ state (*J*-coupling in Fig. 1).

### Incorporation into a polycyclic conjugated hydrocarbon

Once we have identified the best link mode for efficient S_3_ fluorescence in biazulenes, we explore different forms to embed it into a PCH while preserving its structure. The motivation for surrounding the biA1 unit with conjugated rings is twofold: (i) ensure the antiparallel planar disposition of the two azulenes by blocking inter-azulene rotations, and (ii) enforce molecular rigidity to hinder secondary non-radiative decay pathways. Moreover, to maintain the predicted properties for biA1, the desired extended form must preserve the symmetry between the two azulene moieties and avoid orbital mixing, *i.e.*, ensure that frontier molecular orbitals (FMOs) are localized on the biazulene unit.

**Fig. 4:**
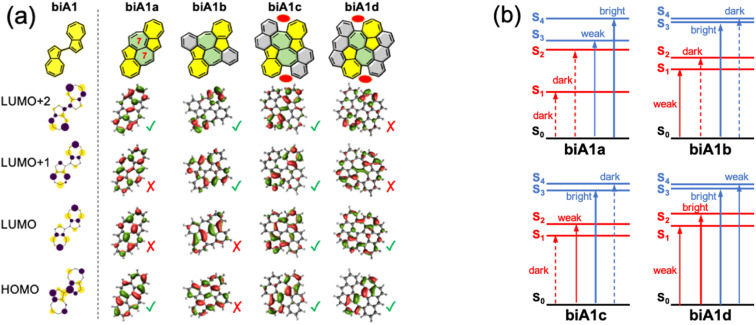
FMOs (a) and state energy diagrams (b) of compounds biA1a–d computed at the rCAM-B3LYP/cc-pVDZ level. Molecular orbitals similar with the (Hückel) biA1 model distribution (left) marked with green checkmarks, otherwise marked with red crosses. Red ovals in structures biA1c and biA1d indicate steric hindrance.

Based on biA1, we can bridge the two azulenes with six-membered or seven-membered rings, which are generally used and easily synthesized. However, when the two azulenes are bridged with two six-membered rings, the FMOs delocalize beyond the biazulene unit and eventually disrupt the properties of biA1 (Fig. S1–S4 and Tables S1–S4†). Then, we propose two seven-membered rings bridged molecules with different number of benzene rings symmetrically fused in order to preserve equivalency between the two azulenes (biA1a–biA1d in Fig. 4a).

The presence of the additional fused rings notably alters the nature of FMOs for biA1a and biA1b with respect to the biA1 model. These changes can be related to the interaction of the biazulene with the peripheral p_*z*_-orbitals, resulting in orbital hybridization and delocalization. Consequently, computed transitions for biA1a (Fig. 4b) discard it as a potentially efficient backbone (weak emission properties of S_3_ and small S_2_/S_3_ gap). On the other hand, despite the different orbital distribution with respect to biA1, biA1b shows good potential capabilities as a high state emitter, with large S_2_/S_3_ gap and oscillator strength. To our interest and delight, the FMOs of biA1c are notably localized on the biazulene core and recover the biA1 model orbital distribution. TDDFT computed low-lying states of biA1c predict good anti-Kasha emission properties from S_3_, with a rather large energy difference with respect to S_2_ and an oscillator strength notably larger than in azulene. It is also worth noticing that the additional benzene rings in biA1c with respect to biA1b, introduce steric hindrance at the armchair region (red ovals in Fig. 4a), triggering the loss of planarity through molecular torsion, and inducing a decoupling of the biazulene core to the π-system of the external naphthalene moieties, which helps to the localization of FMOs. Extending the peripheral conjugation in biA1d stabilizes the bonding interaction of azulene LUMO+1s (LUMO+2 in biA1 model), swapping the LUMO+1/LUMO+2 energy ordering with respect to biA1c. Consequently, biA1d exhibits the same state distribution as biA1c, but with a much-reduced S_2_/S_3_ gap, induced by the orbital energies of LUMO+1 and LUMO+2. From these results, we conclude that molecular structure based on biA1c can be a good candidate as anti-Kasha (S_3_) emitter.

The addition of benzenoid rings surrounding the biazulene core in biA1b–d with respect to biA1a decreases the local antiaromatic character of the seven-membered rings. Concretely, NICS_zz_(1) results indicate a large release of antiaromaticity with the two more external seven-membered rings becoming non-aromatic (Fig. S11†).

### Experimental realization of the anti-Kasha emitter

**Fig. 5:**
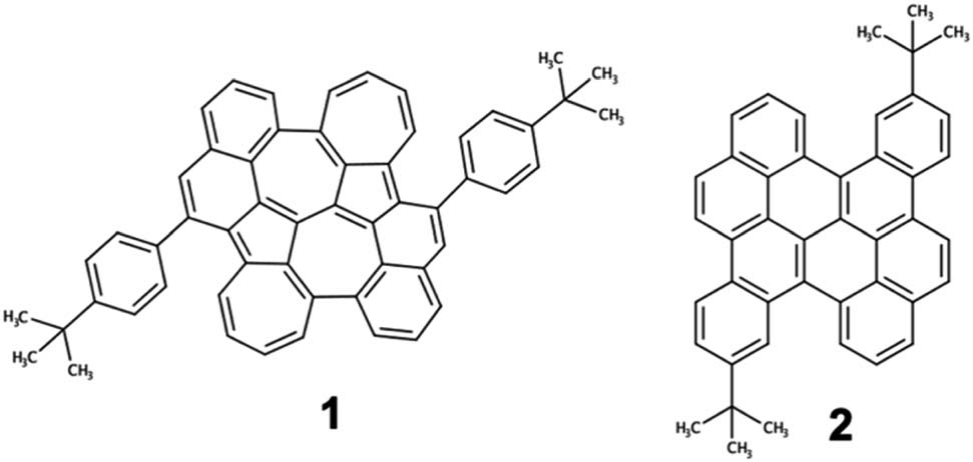
Molecular structures of diazuleno[2,1,8-*efg*:2′,1′,8′-*kla*]heptalene PCH 1 and substituted bischrysene (2).

To prove our strategy, we explore the photophysical properties of the recently synthesized compound 1 (Fig. 5),^51^ corresponding to the optimal biA1c backbone decorated with two *tert*-butylphenyl groups symmetrically distributed. For the sake of completeness, we will compare the photophysical properties of 1 with those of its isoelectronic homologue bischrysene (2), only containing six-membered rings.

**Fig. 6:**
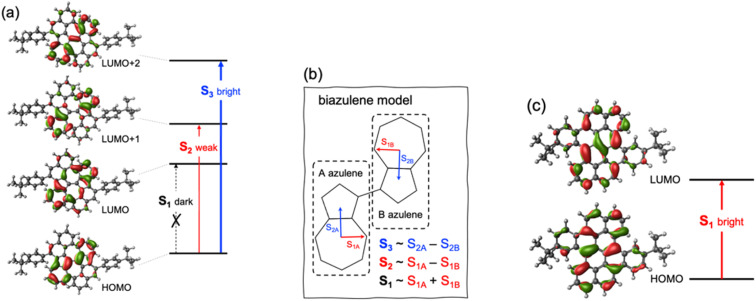
(a) Molecular orbitals involved in the lowest singlet excited states of 1; (b) biazulene (core) model employed to characterize S_1_–S_3_ states of 1; and (c) molecular orbitals involved in the lowest singlet excited state of 2.

The ground state optimized geometry of 1 is noticeably non-planar with bond length alternation along the two embedded azulene units (Table S17†), in very good agreement with the X-ray molecular structure.^51^ FMOs of 1 (Fig. 6a) are largely localized on the biazulene unit with the HOMO built as the in-phase (with respect to inversion) coupling of two monomeric HOMOs on each of the two azulenes, the LUMO (LUMO+1) is the in-phase (out-of-phase) combination of azulene LUMOs, and the LUMO+2 is obtained as the out-of-phase interaction of azulene's LUMO+1. As a result, S_1_ and S_2_ states of 1 can be interpreted as emerging from the + and − combinations of excitations localized on each azulene moiety, respectively (Fig. 6b). The weak inter-azulene coupling results in small S_1_/S_2_ energy gap (*E*(S_1_) = 2.13 eV, *E*(S_2_) = 2.37 eV). Due to the relative disposition of the two azulenes in 1, transition dipole moments in S_1_ cancel each other out, while they present a weak intensity in S_2_ (*f* = 0.068). Interestingly, excitation to S_3_, obtained as the HOMO → LUMO+2 promotion, appears at much larger energy (*E*(S_3_) = 3.20 eV). Moreover, the parallel disposition of local transition dipole moments in the third excited singlet triggers a rather strong oscillator strength (*f* = 0.175). Higher singlet state excitations present much larger delocalization degree over the entire PCH (Fig. S10 and Table S11†). Therefore, the state distribution exhibited by 1 at the Franck–Condon region is in perfect accordance with the simple designed principles stablished from biA1, and the nature of molecular orbitals and low-lying states are in excellent agreement with the biA1c model system (Fig. 4b). On the other hand, the lowest singlet–singlet transition in 2 corresponds to the electronic excitation between the π-delocalized HOMO and LUMO (Fig. 6c). The vertical excitation to S_1_ is computed at 3.07 eV with a large oscillator strength (*f* = 0.442), in agreement with the conventional behavior of other PCHs. Overall, these results are in perfect agreement with the experimental absorption profiles of 1 and 2.^51^

**Fig. 7:**
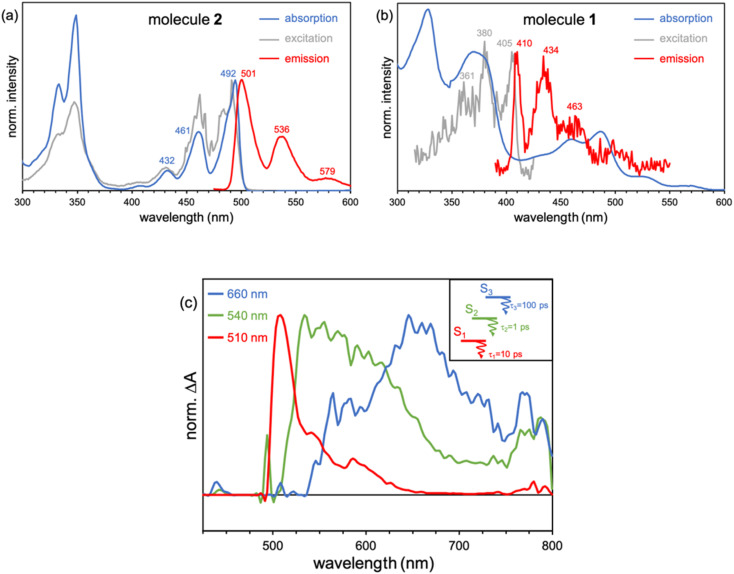
(a) Absorption (black), excitation (gray) and emission (red line) (exciting at 360 nm) spectra of 2 in methyl THF at 80 K. (b) Absorption (black) of 1 together with its emission spectrum (red, exciting at 360 nm) and the corresponding excitation (grey) spectrum of this emission at 80 K. (c) Femtosecond excited state transient absorption spectra (ESA bands) of the species formed upon excitation at 370 nm in methyl THF solution at room temperature together with the excited state diagram and lifetimes deduced from the experimental photophysical data for 1.

Recorded steady-state fluorescence emission spectra of 2 displays one red-shifted band with respect to the S_0_ → S_1_ absorption, which is mirror-like image with the excitation spectrum with a small Stokes shift, indicating the rigidity of its PCH skeleton (Fig. 7a). 2 strictly follows Kasha's rule, that is, independently of the frequency of absorbed photons, highly efficient S_*n*_ → S_1_ IC yields strong S_1_ population and posterior emission. On the other hand, 1 discloses completely different photophysics. When 1 is excited at either one of the two low-energy absorption bands, *i.e.*, 600–800 nm and 430–500 nm, no fluorescence emission is observed either at room temperature or upon temperature lowering at 80 K. Excitation on the high energy group of bands (370 nm) of 1 gives rise to no neat emission, whereas a well-vibronically resolved emission band is clearly detected upon cooling at 80 K, with vibrational peaks at 410, 434 and 463 nm (Fig. 7b), and associated lifetime of 2–3 ns. The excitation spectrum recorded for this emission is also a specular image of its emission, also with vanishing Stokes shift. However, the excitation spectrum does not perfectly fit the absorption profile though the main excitation peaks (at 361, 380 and 405 nm) lie within the limits of its corresponding absorption band (340–410 nm). Though the compound is prepared with a high degree of purity,^51^ the presence of trace amounts of impurities might give rise to anomalous emissions and wrong anti-Kasha assignments. However, here, the presence of a turning on of the fluorescence at cryogenic conditions points out towards the existence of an intramolecular process ruling out the presence of other molecular entities. TDDFT calculations characterize this emission as the S_3_ → S_0_ electronic deexcitation (Tables S14 and S15†) and, according to its vibronic resolution, it belongs to a rigidified S_3_ state of 1 highly prompted to fluoresce.

Femtosecond transient absorption spectroscopy (fs-TAS) of 1 (Fig. 7c) has been carried out to further characterize the manifold of excited states detected in the steady-state emission. Upon pumping at 370 nm, we detect the initial formation of three different excited state absorption bands (ESA) with wavelength absorbance maxima at 660, 540 and 510 nm (Fig. S12†). The band component at 540 nm disappears in 1 ps followed by the ESA of the 510 nm band in 10 ps. Lastly, the 660 nm band gets to zero absorbance in 100 ps. On the other hand, by photoexcitation at 480 nm, only two components in the band time evolution are observed corresponding to the ESA features at 540 and 510 nm. The formation of the ESA feature at 660 nm is only under 370 nm excitation, which, according to TDDFT calculations and assignment, might indicate that corresponds to an absorption from the S_3_ high energy state, *i.e.*, S_3_ → S_*n*_, whereas those ESA bands at 540 and 510 nm emerge as transient absorptions from the two lowest energy lying excited states, *i.e.*, S_2_ → S_*n*_ and S_1_ → S_*n*_, respectively. The observed 660 → 540 → 510 nm blue-shift also supports the excited state energy ordering as: S_3_ (660 nm) > S_2_ (540 nm) > S_1_ (510 nm). On the other hand, the larger relaxation time for the higher energy S_3_ (660 nm) excited state fully agrees with the detection of its fluorescence in the steady state experiment and by electronic structure calculations (see above). On the other hand, the fs-TAS characterization of 2 gave very different results (Fig. S13†). As seen, upon low energy excitation (485 nm, matching the low energy absorption band), a band centered at 710 nm is formed right upon the technique time detection limit. This band is assigned to an excited singlet state that decays to ground state with a lifetime of 800 ps, in accordance with the obtained fluorescence lifetime for 2 (Fig. S14†).

In order to rationalize the high energy anti-Kasha emission of 1, we computationally search for local energy minima on the excited state manifold. Relaxation on the PES of the S_1_ (dark) state substantially reduces the vertical gap to S_0_ (Table S12†), facilitating the non-radiative decay to the ground state. Minimization of the S_2_ energy preserves its electronic character with a weak oscillator strength (Table S13†). Moreover, at the S_2_ minimum, there is a rather small S_2_/S_1_ vertical gap, suggesting fast IC to S_1_ with a subsequent (non-radiative) transit to the ground state. These results are consistent with the lack of fluorescent emission when the molecule is photoactivated with low-energy photons, and with the fast decay of the ESA bands at 540 and 510 nm. This behavior can be rationalized by: (i) weak absorption to low-lying excited states (S_1_ and S_2_); (ii) fast IC to the S_1_ triggered by the small energy gap; and (iii) reduced S_1_/S_0_ optical gap favoring non-radiative decay to the ground state over fluorescent emission.

On the other hand, our TDDFT calculations identify a minimum on the PES of the S_3_ (bright) state (Table S14†), with a molecular geometry that slightly deviate from the Franck–Condon structure (Table S17†). The computed energy at the excited state structure is compatible with the anti-Kasha fluorescence registered at 410 nm and shows large oscillator strength, facilitating their radiative decay. Moreover, it presents a quite large energy gap with lower states of the same multiplicity, *i.e.*, S_2_ and S_1_. In particular, S_3_/S_2_ vertical gap at the S_3_ minimum is considerably large (∼0.8 eV), inhibiting IC to S_2_ (and S_1_).

From these results, we can stablish that the anti-Kasha emission in 1 has its origin in the photophysical properties of azulene. Concretely, the nature of the low-lying singlet states of 1 are largely dictated by the interaction of the two azulene moieties in the biazulene core, resulting in two dark (or weakly emitting) singlets reminiscent of the lowest excited singlet in azulene, and a higher (S_3_) state with important optical activity (related to the second singlet of azulene). Although in 1 the gap of the S_3_ state with the lower singlets is reduced with respect to the S_1_/S_2_ energy difference in pristine azulene (Table S15†), it remains sufficiently large for the emission purpose. On the other hand, the parallel and constructive *J*-like disposition of the two local (azulene-like) transition dipole moments composing the S_3_ → S_0_ transition, triggers a notably stronger oscillator strength as compared to the non-Kasha state in azulene. Moreover, the molecular structure rigidity imposed by the embedding of the biazulene core in a PCH hinders vibrational distortions potentially assisting the deactivation of the high energy singlets.

## Conclusions

In summary, we propose a molecular strategy for intense anti-Kasha emission referring to the intramolecular *J*-coupling concept. The key strategy is to optimize FMO coupling and energy level alignment to selectively increase the photoactivity of higher-order excited states (S_3_), while rationally controlling energy gaps between different excited states to suppress IC processes. To validate our strategy, we have explored the emission properties of the recently synthetized diazuleno[2,1,8-*efg*:2′,1′,8′-*kla*]heptalene embedded in a PCH holding the optimal connection mode of biazulene and the surrounding fused units as computationally predicted. Contrary to its benzenoid isomer 2 only exhibiting luminescence following Kasha's rule, PCH 1 shows fluorescence from the S_3_ state in the range of 410–470 nm upon excitation at 370 nm, which was well verified by fs-TAS, as corresponding to high energy excited state absorption bands at 660 nm. Our work reported herein provides a new way to rationally design anti-Kasha emitters and encourages the exploration of improving the competition of emission against nonradiative decay at high-energy states of photoluminescent materials. New strategies of manipulation of light–matter interaction in organic molecules revealed from these sort of in-depth multidisciplinary analysis paves the unique way for rational design of new chromophores and energy converter in all-carbon-based molecular systems.

## Data availability

The datasets supporting this article have been uploaded as part of the ESI.†

## Author contributions

A. D.-A. performed all the electronic structure calculations. A. D.-A. and D. C. analyzed the computational results. J. M.-B., J. W., J. L. and J. C. performed experiments for molecules 1 and 2. J. M.-B. and J. C. carried out transient and steady-state spectroscopic measurements and discuss the interpretation. J. L., J. C. and D. C. conceived the work and wrote the paper.

## Conflicts of interest

There are no conflicts to declare.

## Supplementary Material

SC-014-D3SC00405H-s001
