# Supplementary material for: Rational design of anti-Kasha photoemission from a biazulene core embedded in an antiaromatic/aromatic hybrid
Source: Chem Sci. 2023 May 22;14(23):6420–9. doi: 10.1039/d3sc00405h (PMC10266467; doi:10.1039/d3sc00405h)
Supplement: SC-014-D3SC00405H-s001 [file SC-014-D3SC00405H-s001.pdf]

Electronic Supplementary Material (ESI) for Chemical Science.
This journal is © The Royal Society of Chemistry 2023

Supporting Information for:

Rational design of anti-Kasha photoemission from a biazulene core embedded in an antiaromatic/aromatic hybrid

Aitor Diaz-Andres¶, Jose Marín-Beloqui‡, Junting Wang†, Junzhi Liu†, Juan Casado‡, David Casanova¶,#

¶Donostia International Physics Center (DIPC), 20018 Donostia, Euskadi, Spain

†Department of Chemistry and State Key Laboratory of Synthetic Chemistry, The University of Hong Kong, Pokfulam Road, Hong Kong, China

‡Department of Physical Chemistry, University of Malaga Campus de Teations s/n, 229071 Malaga, Spain

¶Donostia International Physics Center (DIPC), 20018 Donostia, Euskadi, Spain

#IKERBASQUE − Basque Foundation for Science, 48009 Bilbao, Euskadi, Spain

CONTENTS

1. Electronic structure calculations

Hückel model

Molecular orbitals and energies of biA1 homologues bridged with hexagons

Molecular orbitals at the rCAM-B3LYP level

Vertical excitations

Nucleus-independent chemical shift (NICS)

2. Transient absorption and fluorescence measurements

3. Optimized coordinates

1. Electronic structure calculations

Hückel model

Hückel orbital energies were obtained using the orbital parameters α (the energy of an electron in a p-orbital) and β (the interaction energy between two orbitals) from the reported literature [*J. Org. Chem.* **45**, 4801-4802 (1980)].

Molecular orbitals and energies of biA1 homologues bridged with hexagons

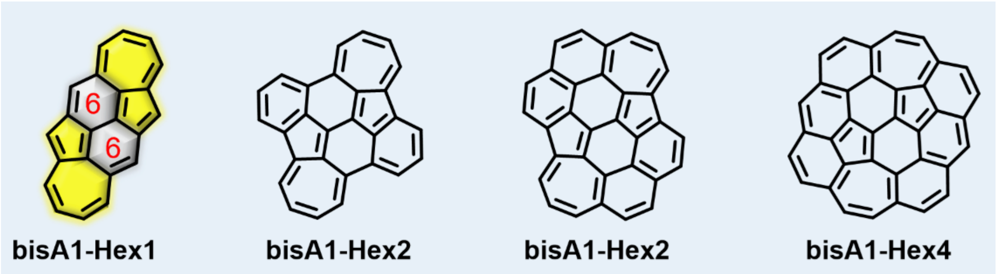

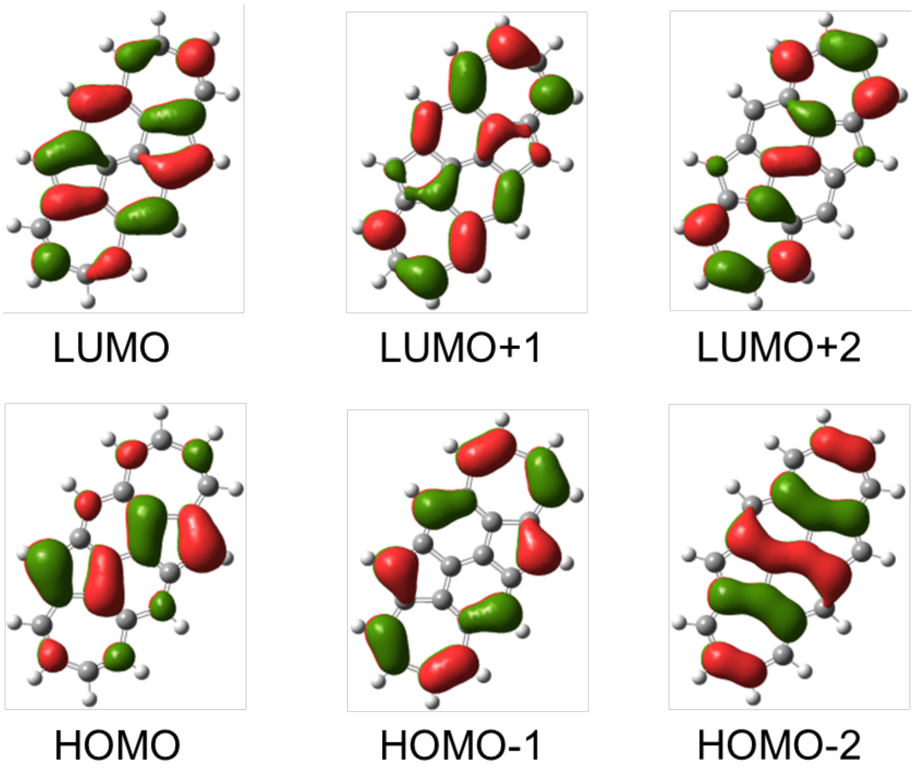

Figure S1. Frontier molecular orbitals of **biA1-Hex1** computed at the B3LYP/6-311G(d,p) level.

Table S1. Vertical excitations (ΔE in eV), oscillation strengths (f), orbital composition and transition dipole moments (μ in a.u.) of low-lying states of **biA1-Hex1** at the Franck-Condon region computed at the B3LYP/6-311G(d,p) level. (H_0, H_1 and H_2 present HOMO, HOMO+1, HOMO+2 orbitals, respectively. L_0, L_1, and L_2 are corresponding to LUMO, LUMO+1, LUMO+2 orbitals, respectively).

State	ΔE	f	Composition	μ
S_1	1.7329	0.0000	$H_0 \to L_0$ (89%);<br>$H_0 \to L_1$ (11%)	0.00 X; 0.00 Y; 0.00 Z
S_2	2.0538	0.0000	$H_0 \to L_0$ (11%);<br>$H_0 \to L_1$ (89%)	0.00 X; 0.00 Y; 0.00 Z
S_3	2.5719	0.0556	$H_1 \to L_0$ (62%);<br>$H_0 \to L_2$ (25%);<br>$H_1 \to L_1$ (7%)	0.90 X; –0.26 Y; 0.00 Z
S_4	2.8320	0.0612	$H_1 \to L_1$ (74%);<br>$H_1 \to L_0$ (17%);<br>$H_2 \to L_0$ (5%)	–0.31 X; –0.89 Y; 0.00 Z
S_5	3.0602	0.0801	$H_0 \to L_2$ (61%);<br>$H_2 \to L_1$ (12%);<br>$H_0 \to L_0$ (12%)	–0.96 X; 0.38 Y; 0.00 Z

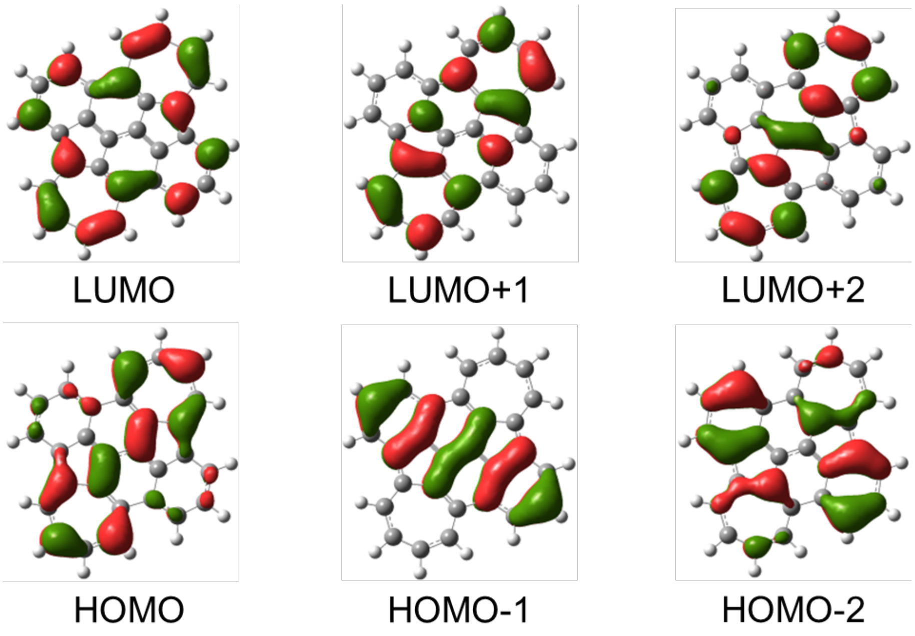

Figure S2. Frontier molecular orbitals of **biA1-Hex2** computed at the B3LYP/6-311G(d,p) level.

Table S2. Vertical excitations (ΔE in eV), oscillation strengths (f), orbital composition and transition dipole moments (μ in a.u.) of low-lying states of **biA1-Hex2** at the Franck-Condon region computed at the B3LYP/6-311G(d,p) level. (H_0, H_1, H_2 and H_4 present HOMO, HOMO+1, HOMO+2, HOMO+4 orbitals, respectively. L_0, L_1, L_2 and L_3 are corresponding to LUMO, LUMO+1, LUMO+2, LUMO+3 orbitals, respectively).

State	ΔE	f	Composition	μ
**S_1**	0.9602	0.0252	$H_0 \rightarrow L_0$ (95%);<br>$H_0 \rightarrow L_2$ (5%)	0.91 X; 0.49 Y; 0.00 Z
**S_2**	1.3144	0.0000	$H_0 \rightarrow L_1$	0.00 X; 0.00 Y; 0.00 Z
**S_3**	2.3807	0.3152	$H_0 \rightarrow L_2$ (87%);<br>$H_2 \rightarrow L_0$ (6%);<br>$H_0 \rightarrow L_0$ (5%)	–2.29 X; 0.43 Y; 0.00 Z
**S_4**	2.6470	0.0000	$H_1 \rightarrow L_0$ (95%);<br>$H_0 \rightarrow L_3$ (5%);	–0.00 X; –0.00 Y; 0.00 Z
**S_5**	2.8552	0.0000	$H_0 \rightarrow L_3$ (91%);<br>$H_1 \rightarrow L_0$ (4%);<br>$H_4 \rightarrow L_0$ (4%)	–0.00 X; 0.00 Y; 0.00 Z

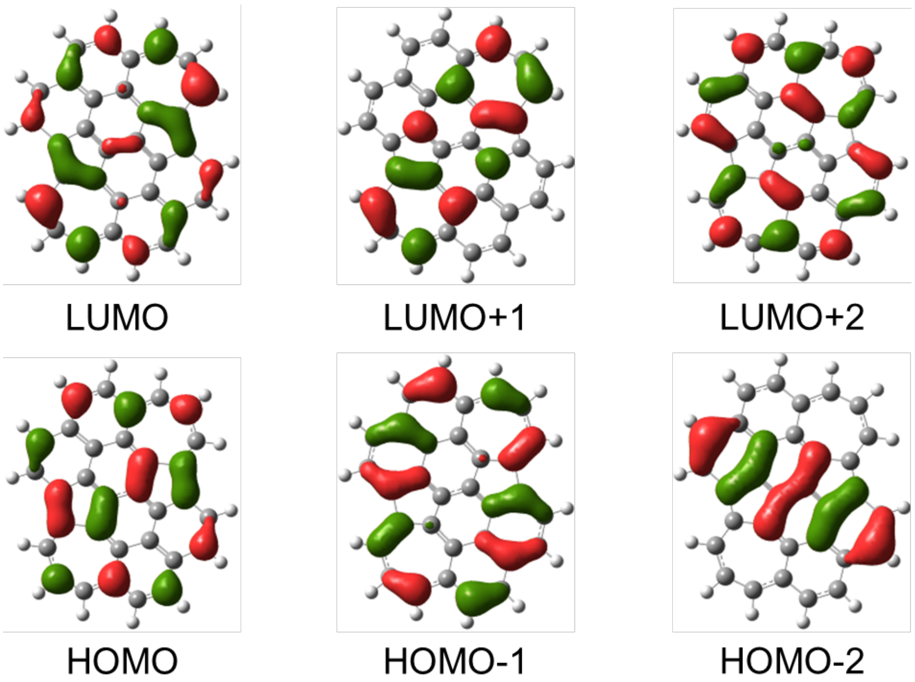

Figure S3. Frontier molecular orbitals of **biA1-Hex3** computed at the B3LYP/6-311G(d,p) level.

Table S3. Vertical excitations (Δ*E* in eV), oscillation strengths (*f*), orbital composition and transition dipole moments (μ in a.u.) of low-lying states of **biA1-Hex3** at the Franck-Condon region computed at the B3LYP/6-311G(d,p) level. (H_0, H_1, H_2 and H_3 present HOMO, HOMO+1, HOMO+2, HOMO+3 orbitals, respectively. L_0, L_1 and L_2 are corresponding to LUMO, LUMO+1, LUMO+2 orbitals, respectively.)

State	**Δ*E***	*f*	Composition	μ
**S_1**	1.2433	0.0182	$H_0 \rightarrow L_0$ (97%)<br>$H_0 \rightarrow L_2$ (3%)	0.34 X; –0.70 Y; 0.00 Z
**S_2**	1.5663	0.0000	$H_0 \rightarrow L_1$	0.00 X; 0.00 Y; 0.00 Z
**S_3**	2.1071	0.1366	$H_0 \rightarrow L_2$ (79%)<br>$H_1 \rightarrow L_0$ (19%)<br>$H_0 \rightarrow L_0$ (2%)	–1.63 X; 0.05 Y; 0.00 Z
**S_4**	2.8419	0.0000	$H_2 \rightarrow L_0$ (%)	0.00 X; 0.00 Y; 0.00 Z
**S_5**	2.9415	0.0000	$H_1 \rightarrow L_0$ (74%)<br>$H_0 \rightarrow L_2$ (14%)<br>$H_3 \rightarrow L_1$ (3%)	–2.55 X; –0.56 Y; 0.00 Z

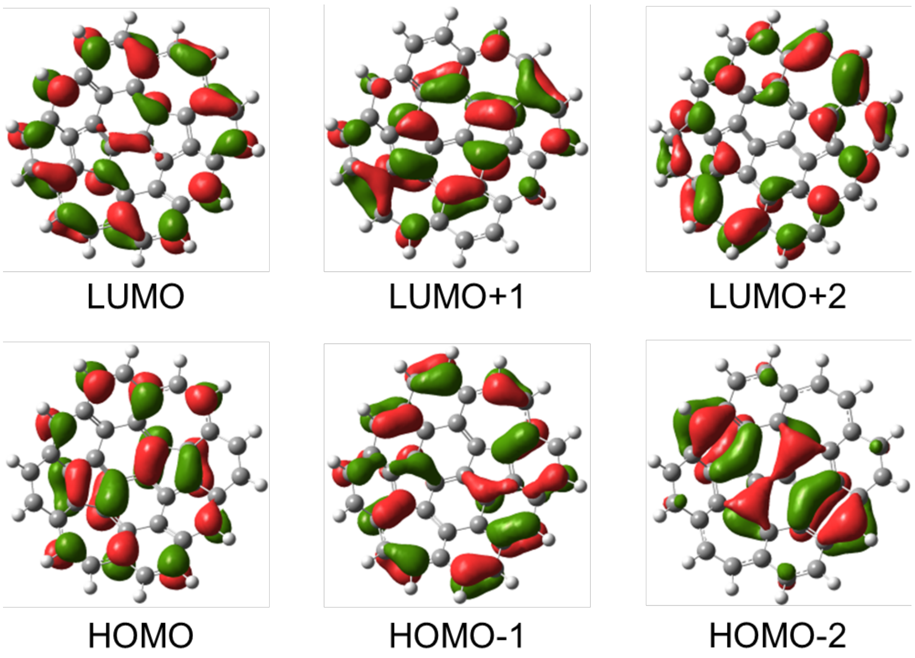

Figure S4. Frontier molecular orbitals of **biA1-Hex4** computed at the B3LYP/6-311G(d,p) level.

Table S4. Vertical excitations (ΔE in eV), oscillation strengths (f), orbital composition and transition dipole moments (μ in a.u.) of low-lying states of **biA1-Hex3** at the Franck-Condon region computed at the B3LYP/6-311G(d,p) level. (H_0, H_1, H_2 and H_3 present HOMO, HOMO+1, HOMO+2, HOMO+3 orbitals, respectively. L_0, L_1 and L_2 are corresponding to LUMO, LUMO+1, LUMO+2 orbitals, respectively).

State	ΔE	f	Composition	μ
S_1	1.2913	0.0003	$H_0 \rightarrow L_1$	0.00 X; 0.00 Y; 0.09 Z
S_2	1.5532	0.0780	$H_0 \rightarrow L_0$ (88%)<br>$H_0 \rightarrow L_2$ (12%)	–0.58 X; 1.31 Y; 0.00 Z
S_3	1.9579	0.0567	$H_0 \rightarrow L_2$ (86%)<br>$H_0 \rightarrow L_0$ (10%)<br>$H_0 \rightarrow L_0$ (3%)	–0.85 X; 0.68 Y; 0.00 Z
S_4	2.6643	0.0028	$H_1 \rightarrow L_1$ (98%)<br>$H_3 \rightarrow L_1$ (2%)	0.00 X; 0.00 Y; –0.21 Z
S_5	2.6817	0.1687	$H_1 \rightarrow L_0$ (92%)<br>$H_3 \rightarrow L_2$ (5%)<br>$H_0 \rightarrow L_2$ (2%)	–1.48 X; –0.62 Y; 0.00 Z

Molecular orbitals at the rCAM-B3LYP level

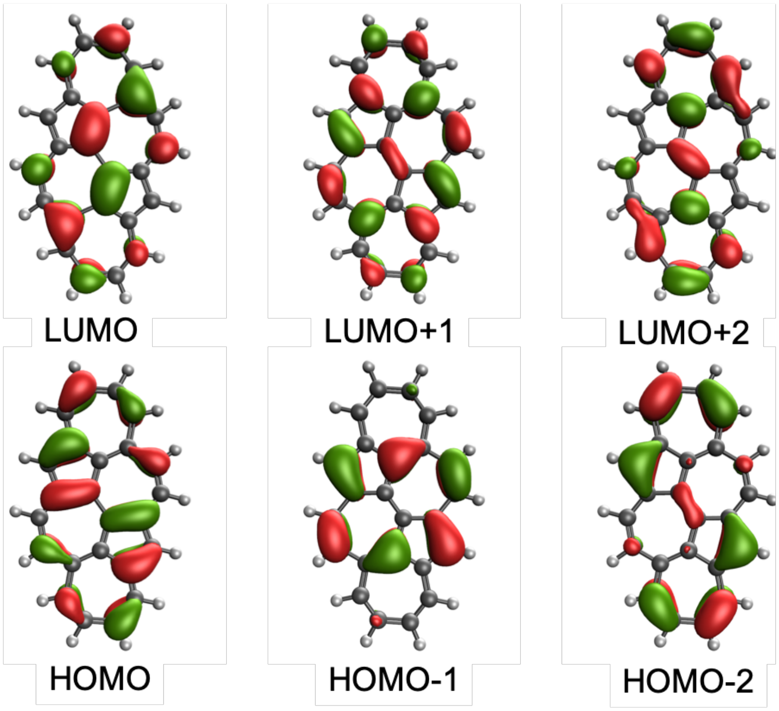

Figure S5. Frontier molecular orbitals of **biA1a** computed at the rCAM-B3LYP/cc-pVTZ level.

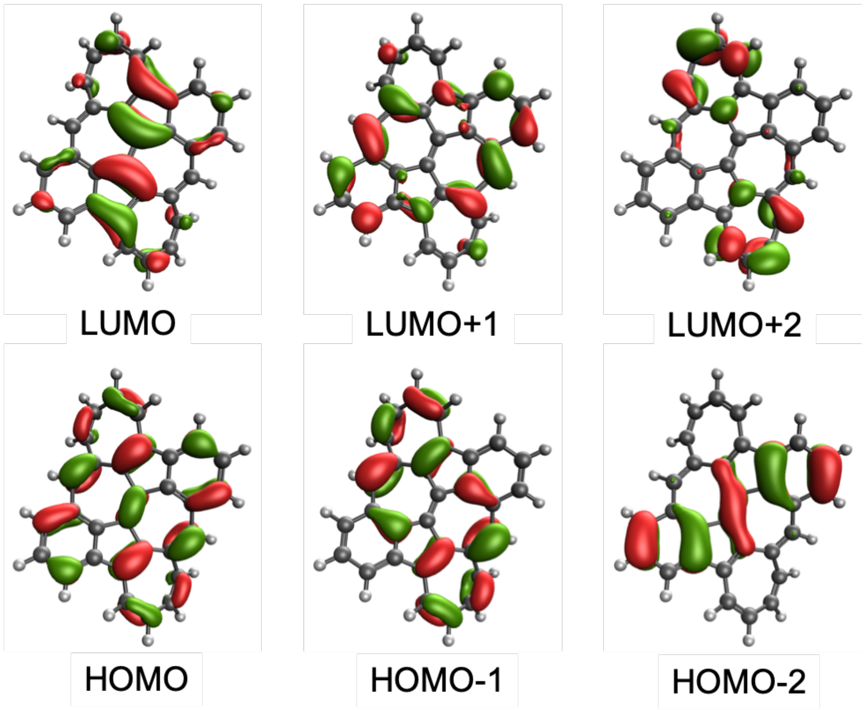

Figure S6. Frontier molecular orbitals of **biA1b** computed at the rCAM-B3LYP/cc-pVTZ level.

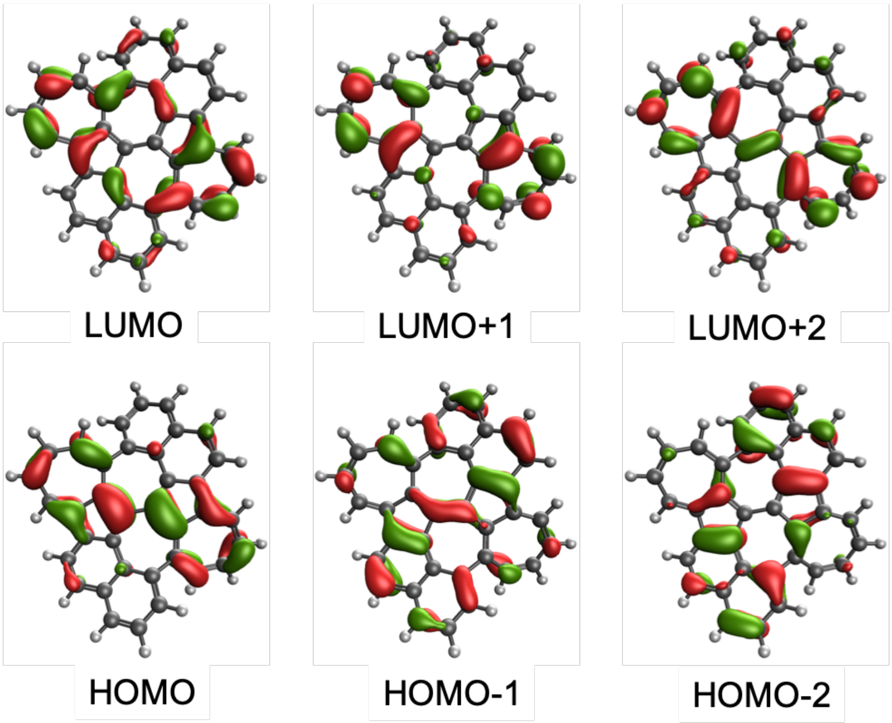

Figure S7. Frontier molecular orbitals of **biA1c** computed at the rCAM-B3LYP/cc-pVTZ level.

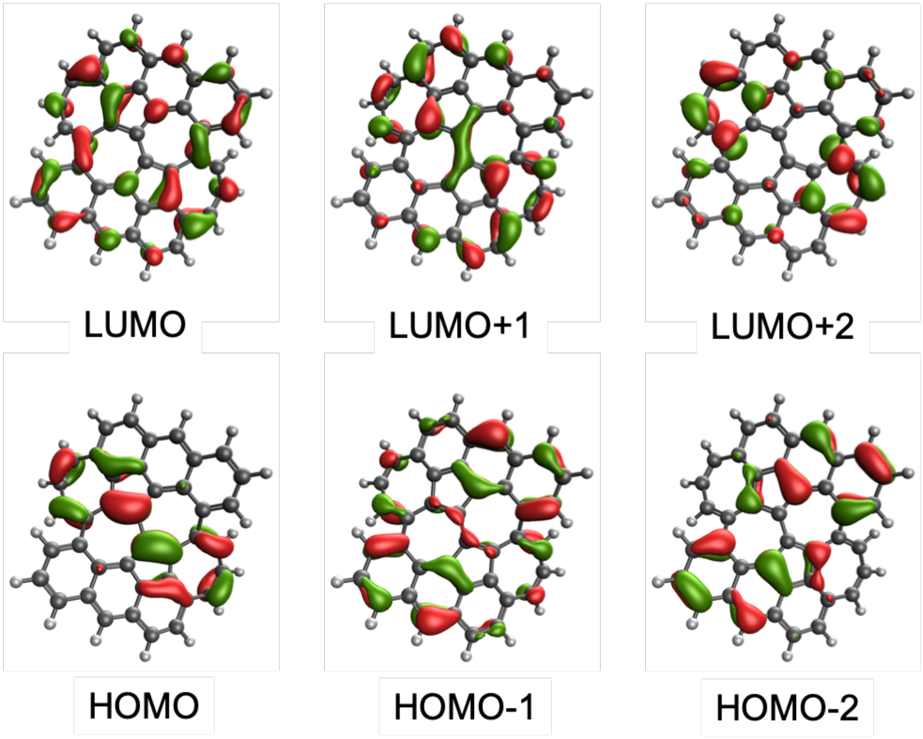

Figure S8. Frontier molecular orbitals of **biA1d** computed at the rCAM-B3LYP/cc-pVTZ level.

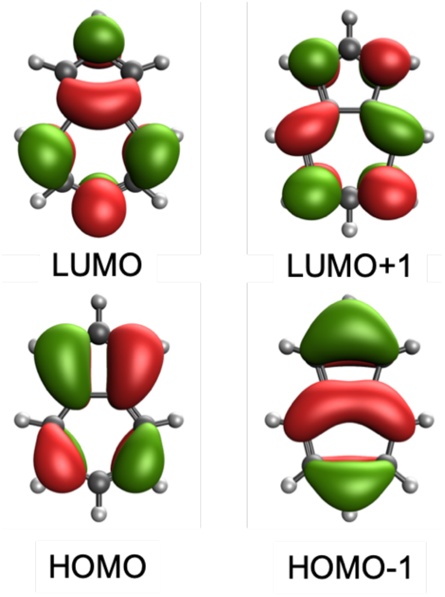

Figure S9. Frontier molecular orbitals of **azulene** at the rCAM-B3LYP/cc-pVTZ level.

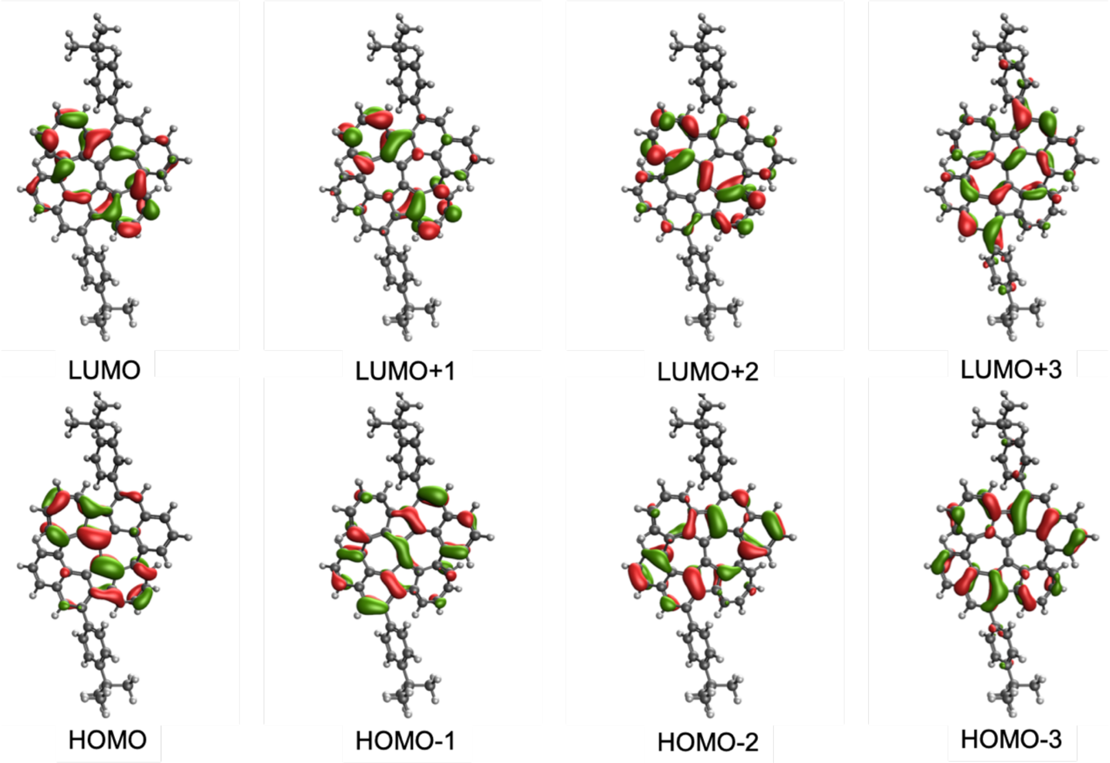

Figure S10. Frontier molecular orbitals of **1** computed at the rCAM-B3LYP/cc-pVTZ level.

Vertical excitations

Excitation energies for the studied molecules have been obtained from ground-state geometries optimized at the CAM-B3LYP/cc-pVDZ level and with electronic transitions obtained with a larger basis set (cc-pVTZ) and with the rCAM-B3LYP functional, which has shown to minimize many-electron self-interaction error in largely conjugated systems [J. Chem. Phys. 126, 191109 (2007)].

Table S5. Vertical excitations (ΔE), oscillation strengths (f), orbital composition and transition dipole moments (μ) of low-lying states of **biA1a** at the Franck-Condon region computed at the rCAM-B3LYP/cc-pVTZ level (H_0, H_1 and H_2 present HOMO, HOMO+1, HOMO+2 orbitals, respectively. L_0, L_1, L_2 and L_3 are corresponding to LUMO, LUMO+1, LUMO+2, LUMO+3 orbitals, respectively).

State	ΔE, eV	f	Composition	Trans. Mom.
S_1	1.213	0.0	$H_0 \rightarrow L_0$	0.00 X ; 0.00 Y ; 0.00 Z
S_2	2.327	0.0	$H_1 \rightarrow L_1$	0.00 X ; 0.00 Y ; 0.00 Z
S_3	2.557	0.0265	$H_0 \rightarrow L_2$ (59 %) ; $H_2 \rightarrow L_0$ (32 %)<br>$H_0 \rightarrow L_1$ (5 %)	-0.62 X ; -0.19 Y ; 0.00 Z
S_4	3.142	0.3554	$H_0 \rightarrow L_1$ (77 %) ; $H_0 \rightarrow L_2$ (9 %)	-2.11 X ; -0.40 Y ; 0.00 Z
S_5	3.462	0.0237	$H_1 \rightarrow L_1$ (39 %) ; $H_0 \rightarrow L_3$ (35 %)<br>$H_2 \rightarrow L_0$ (9 %) ; $H_0 \rightarrow L_1$ (6 %)	0.50 X ; 0.18 Y ; 0.00 Z

Table S6. Vertical excitations (ΔE), oscillation strengths (f), orbital composition and transition dipole moments (μ) of low-lying states of low-lying states of **biA1b** at the Franck-Condon region computed at the rCAM-B3LYP/cc-pVTZ level. (H_0, H_1, H_2 H_3 and H_4 present HOMO, HOMO+1, HOMO+2, HUMO+3 and HOMO+4 orbitals, respectively. L_0, L_1, L_2, L_3 and L_4 are corresponding to LUMO, LUMO+1, LUMO+2, LUMO+3, LUMO+4 orbitals, respectively.)

State	ΔE, eV	f	Composition	Trans. Mom.
S_1	2.015	0.0393	$H_0 \rightarrow L_0$ (86 %) ; $H_1 \rightarrow L_2$ (7 %)	0.76 X ; -0.46 Y ; 0.00 Z
S_2	2.340	0.0003	$H_1 \rightarrow L_0$ (84 %) ; $H_0 \rightarrow L_2$ (9 %)	0.00 X ; 0.00 Y ; 0.07 Z
S_3	3.398	0.2270	$H_0 \rightarrow L_1$ (52 %) ; $H_2 \rightarrow L_0$ (51 %)<br>$H_1 \rightarrow L_3$ (10 %)	1.45 X ; -0.79 Y ; 0.00 Z
S_4	3.486	0.0	$H_1 \rightarrow L_1$ (61 %) ; $H_0 \rightarrow L_3$ (22 %)<br>$H_0 \rightarrow L_2$ (7 %)	0.00 X ; 0.00 Y ; 0.02 Z
S_5	3.884	0.1906	$H_2 \rightarrow L_0$ (43 %) ; $H_0 \rightarrow L_4$ (16 %)<br>$H_1 \rightarrow L_2$ (11 %) ; $H_0 \rightarrow L_1$ (8 %)<br>$H_4 \rightarrow L_0$ (8 %) ; $H_0 \rightarrow L_3$ (4 %)	-1.41 X ; 0.10 Y ; 0.00 Z

Table S7. Vertical excitations (ΔE), oscillation strengths (*f*), orbital composition and transition dipole moments (μ) of low-lying states of low-lying states of **biA1c** at the Franck-Condon region computed at the rCAM-B3LYP/cc-pVTZ level. (H_0, H_1, H_2 and H_3 present HOMO, HOMO+1, HOMO+2, HOMO+3 orbitals, respectively. L_0, L_1, L_2 and L_3 are corresponding to LUMO, LUMO+1, LUMO+2, LUMO+3 orbitals, respectively.)

State	ΔE, eV	f	Composition	Trans. Mom.
S_1	1.989	0.0002	$H_0 \rightarrow L_0$ (85 %) ; $H_1 \rightarrow L_1$ (8 %)	0.00 X ; 0.00 Y ; 0.07 Z
S_2	2.268	0.0627	$H_0 \rightarrow L_1$ (72 %) ; $H_1 \rightarrow L_0$ (19 %)	01.06 X ; -0.01 Y ; 0.00 Z
S_3	3.120	0.1947	$H_0 \rightarrow L_2$ (72 %) ; $H_3 \rightarrow L_0$ (7 %)	-0.89 X ; 1.32 Y ; 0.00 Z
S_4	3.323	0.0053	$H_2 \rightarrow L_0$ (48 %) ; $H_0 \rightarrow L_3$ (17 %)<br>$H_3 \rightarrow L_1$ (8 %) ; $H_1 \rightarrow L_2$ (8 %)	0.00 X ; 0.00 Y ; 0.26 Z
S_5	3.813	0.0430	$H_0 \rightarrow L_3$ (52 %) ; $H_2 \rightarrow L_0$ (26 %)	0.00 X ; 0.00 Y ; 0.68 Z

Table S8. Vertical excitations (ΔE), oscillation strengths (*f*), orbital composition and transition dipole moments (μ) of low-lying states of low-lying states of **biA1d** at the Franck-Condon region computed at the rCAM-B3LYP/cc-pVTZ level. (H_0, H_1, H_2 and H_3 present HOMO, HOMO+1, HOMO+2, HOMO+3 orbitals, respectively. L_0, L_1, L_2 and L_3 are corresponding to LUMO, LUMO+1, LUMO+2, LUMO+3 orbitals, respectively.)

State	ΔE, eV	f	Composition	Trans. Mom.
S_1	1.963	0.0002	$H_0 \rightarrow L_0$ (86 %) ; $H_1 \rightarrow L_2$ (6 %)	0.00 X ; 0.00 Y ; 0.06 Z
S_2	2.270	0.1069	$H_0 \rightarrow L_1$ (51 %) ; $H_0 \rightarrow L_2$ (22 %)<br>$H_1 \rightarrow L_0$ (15 %)	0.06 X ; 1.38 Y ; 0.00 Z
S_3	2.881	0.2596	$H_0 \rightarrow L_2$ (51 %) ; $H_0 \rightarrow L_1$ (29 %)	1.56 X ; 1.12 Y ; 0.00 Z
S_4	3.015	0.0157	$H_2 \rightarrow L_0$ (29 %) ; $H_0 \rightarrow L_3$ (29 %)<br>$H_1 \rightarrow L_1$ (25 %)	0.00 X ; 0.00 Y ; -0.46 Z
S_5	3.317	0.3456	$H_2 \rightarrow L_1$ (39 %) ; $H_1 \rightarrow L_0$ (35 %)<br>$H_3 \rightarrow L_0$ (7 %) ; $H_0 \rightarrow L_1$ (5 %)<br>$H_1 \rightarrow L_3$ (5 %)	2.06 X ; -0.01 Y ; 0.00 Z

Table S9. Vertical excitations (ΔE), oscillation strengths (*f*), orbital composition and transition dipole moments (μ) of low-lying states of low-lying states of **2** at the Franck-Condon region computed at the rCAM-B3LYP/cc-pVTZ level. (H_0, and H_1 present HOMO, HOMO+1 orbitals, respectively. L_0 and L_1 are corresponding to LUMO, LUMO+1, LUMO+2, LUMO+3 orbitals, respectively.)

State	ΔE, eV	f	Composition	Trans. Mom.
S_1	3.073	0.4422	$H_0 \rightarrow L_0$	-0.87 X ; -2.26 Y ; -0.17 Z
S_2	3.661	0.0001	$H_1 \rightarrow L_0$; $H_0 \rightarrow L_1$	-0.03 X ; -0.01 Y ; -0.02 Z

Table S10. Vertical $S_1 \rightarrow S_0$ deexcitation, oscillation strengths (*f*), orbital composition and transition dipole moments (μ) of **2** at the S_1 optimized geometry- computed at the rCAM-B3LYP/cc-pVTZ level. (H_0 and L_0 present HOMO and LUMO orbitals.)

State	ΔE, eV	f	Composition	Trans. Mom.
S_1	2.660	0.4552	$H_0 \rightarrow L_0$	-0.84 X ; -2.50 Y ; -0.17 Z

Table S11. Vertical excitations, oscillation strengths (*f*), orbital composition and transition dipole moments (μ) of low-lying states of **1** at the Franck-Condon region computed at the rCAM-B3LYP/cc-pVTZ level. (H_0, H_1, H_2, H_3 and H_4 present HOMO, HOMO+1, HOMO+2, HOMO+3, HOMO+4 orbitals, respectively. L_0, L_1, L_2, L_3, L_5 and L_6 are corresponding to LUMO, LUMO+1, LUMO+2, LUMO+3, LUMO+5, LUMO+6 orbitals, respectively.)

State	ΔE, eV	f	Composition	
S_1	2.128	0.0008	$H_0 \rightarrow L_0$ (84 %) ; $H_1 \rightarrow L_1$ (8 %)	0.00 X ; 0.01 Y ; -0.12 Z
S_2	2.373	0.0675	$H_0 \rightarrow L_1$ (71 %) ; $H_1 \rightarrow L_0$ (19 %)	0.33 X ; 1.03 Y ; 0.00 Z
S_3	3.198	0.1753	$H_0 \rightarrow L_2$ (69 %) ; $H_3 \rightarrow L_0$ (7 %)<br>$H_2 \rightarrow L_1$ (5 %)	-1.16 X ; 0.95 Y ; 0.00 Z
S_4	3.363	0.0086	$H_2 \rightarrow L_0$ (44 %) ; $H_0 \rightarrow L_3$ (17 %)<br>$H_1 \rightarrow L_2$ (9 %) ; $H_3 \rightarrow L_1$ (8 %)<br>$H_1 \rightarrow L_1$ (7 %)	0.00 X ; 0.00 Y ; -0.32 Z
S_5	3.843	0.0482	$H_0 \rightarrow L_3$ (48 %) ; $H_2 \rightarrow L_0$ (28 %)	0.01 X ; 0.01 Y ; -0.72 Z
S_6	4.037	0.6792	$H_1 \rightarrow L_0$ (26 %) ; $H_2 \rightarrow L_1$ (26 %)<br>$H_0 \rightarrow L_1$ (9 %) ; $H_0 \rightarrow L_4$ (8 %)<br>$H_3 \rightarrow L_0$ (5 %)	2.19 X ; -1.43 Y ; -0.01 Z
S_7	4.111	0.1212	$H_1 \rightarrow L_0$ (22 %) ; $H_2 \rightarrow L_1$ (13 %)<br>$H_3 \rightarrow L_0$ (12 %) ; $H_0 \rightarrow L_2$ (8 %)<br>$H_3 \rightarrow L_3$ (5 %)	0.20 X ; 1.08 Y ; 0.00 Z
S_8	4.214	0.3170	$H_2 \rightarrow L_2$ (27 %) ; $H_2 \rightarrow L_1$ (17 %)<br>$H_1 \rightarrow L_0$ (8 %) ; $H_1 \rightarrow L_5$ (7 %)<br>$H_0 \rightarrow L_6$ (5 %)	-1.23 X ; -1.25 Y ; 0.00 Z
S_9	4.263	0.0157	$H_1 \rightarrow L_1$ (33 %) ; $H_2 \rightarrow L_3$ (11 %)<br>$H_3 \rightarrow L_2$ (9 %) ; $H_1 \rightarrow L_2$ (7 %)<br>$H_0 \rightarrow L_5$ (6 %) ; $H_0 \rightarrow L_0$ (6 %)	-0.01 X ; 0.00 Y ; 0.39 Z

Table S12. Vertical $S_1 \rightarrow S_0$ deexcitation, oscillation strengths (*f*), orbital composition and transition dipole moments (μ) of **1** at the S_1 optimized geometry computed at the rCAM-B3LYP/cc-pVTZ level. (H_0 and L_0 present HOMO and LUMO orbitals.)

State	ΔE, eV	f	Composition	Trans. Mom.
S_1	0.997	0.0000	$H_0 \rightarrow L_0$ (91 %) ; $H_1 \rightarrow L_1$ (5 %)	0.00 X ; 0.01 Y ; -0.04 Z

Table S13. Vertical excitations, oscillation strengths (*f*), orbital composition and transition dipole moments (μ) of **1** at the S_2 optimized geometry computed at the rCAM-B3LYP/cc-pVTZ level. (H_0 and H_1 present HOMO and HOMO+1 orbitals, respectively. L_0 and L_1 are corresponding to LUMO and LUMO+1 orbitals, respectively.)

State	ΔE, eV	f	Composition	Trans. Mom.
S_1	1.199	0.0002	$H_0 \rightarrow L_0$ (89 %) ; $H_1 \rightarrow L_1$ (7 %)	0.00 X ; 0.01 Y ; -0.08 Z
S_2	1.545	0.0202	$H_0 \rightarrow L_1$ (78 %) ; $H_1 \rightarrow L_0$ (13 %)	0.42 X ; 0.60 Y ; 0.00 Z

Table S14. Vertical $S_n \rightarrow S_0$ deexcitation, oscillation strengths (*f*), orbital composition and transition dipole moments (μ) of **1** at the S_3 optimized geometry computed at the rCAM-B3LYP/cc-pVTZ level. (H_0 and H_1 present HOMO and HOMO+1 orbitals, respectively. L_0, L_1, L_2 and L_4 are corresponding to LUMO, LUMO+1, LUMO+2, LUMO+4 orbitals, respectively.)

State	ΔE, eV	f	Composition	Trans. Mom.
S_1	1.483	0.0000	$H_0 \rightarrow L_0$ (88 %)	0.00 X ; 0.00 Y ; -0.02 Z
S_2	1.917	0.0315	$H_0 \rightarrow L_2$ (46 %) ; $H_0 \rightarrow L_1$ (29 %) $H_1 \rightarrow L_0$ (18 %)	-0.51 X ; -0.64 Y ; 0.00 Z
S_3	2.668	0.2966	$H_0 \rightarrow L_1$ (56 %) ; $H_0 \rightarrow L_2$ (25 %)	1.49 X ; -1.52 Y ; 0.00 Z

Table S15. Vertical excitations, oscillation strengths (*f*), orbital composition and transition dipole moments (μ) of low-lying states of azulene at the Franck-Condon region computed at the rCAM-B3LYP/cc-pVTZ level. (H_0 and H_1 present HOMO and HOMO+1 orbitals, respectively. L_0 and L_1 are corresponding to LUMO and LUMO+1 orbitals, respectively.)

State	ΔE, eV	f	Composition	Trans. Mom.
S_1	2.378	0.0076	$H_0 \rightarrow L_0$	-0.36 X ; 0.00 Y ; 0.00 Z
S_2	3.806	0.0026	$H_1 \rightarrow L_0$; $H_0 \rightarrow L_1$	0.00 X ; 0.00 Y ; -0.17 Z

Nucleus-independent chemical shift (NICS)

NICS values [J. Am. Chem. Soc. 118, 6317-6318 (1996)] for the studied molecules have been computed at the rCAM-B3LYP/cc-pVTZ level at the optimized ground-state geometries.

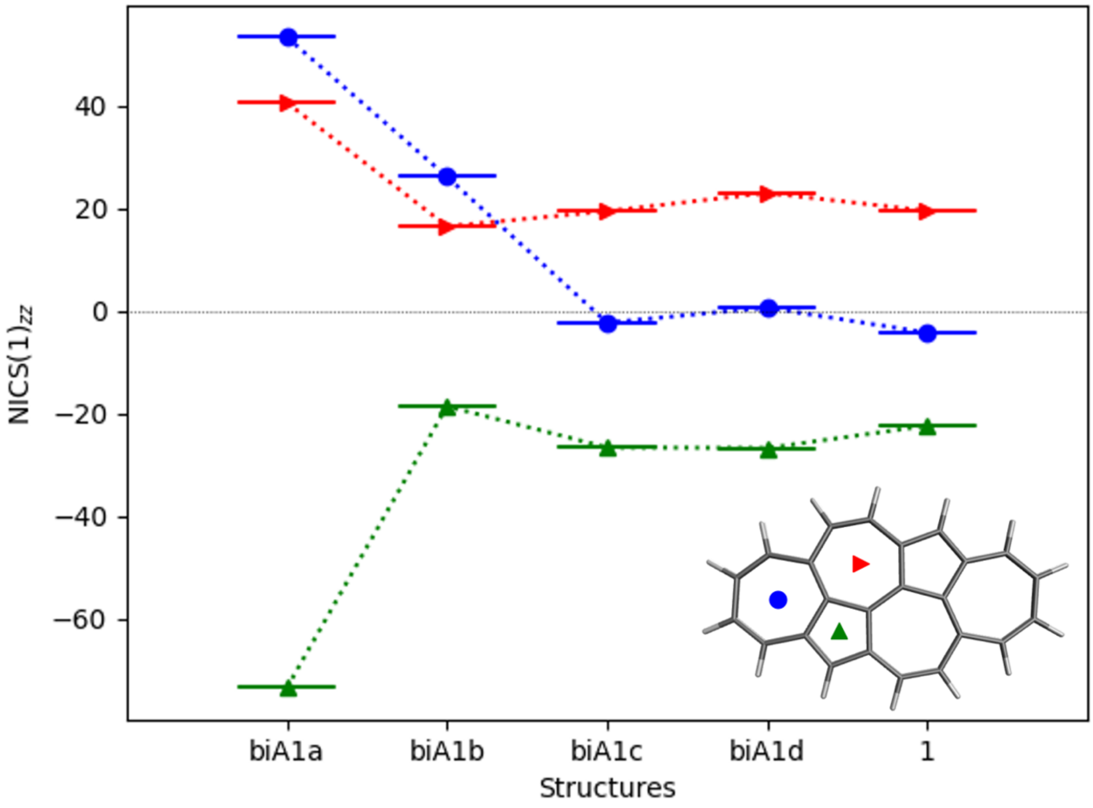

Figure S11. Ground state $NICS_{zz}(1)$ at the center of five- and seven-membered rings of compounds **biA1a-d** and **1**.

2. Transient absorption and fluorescence measurements

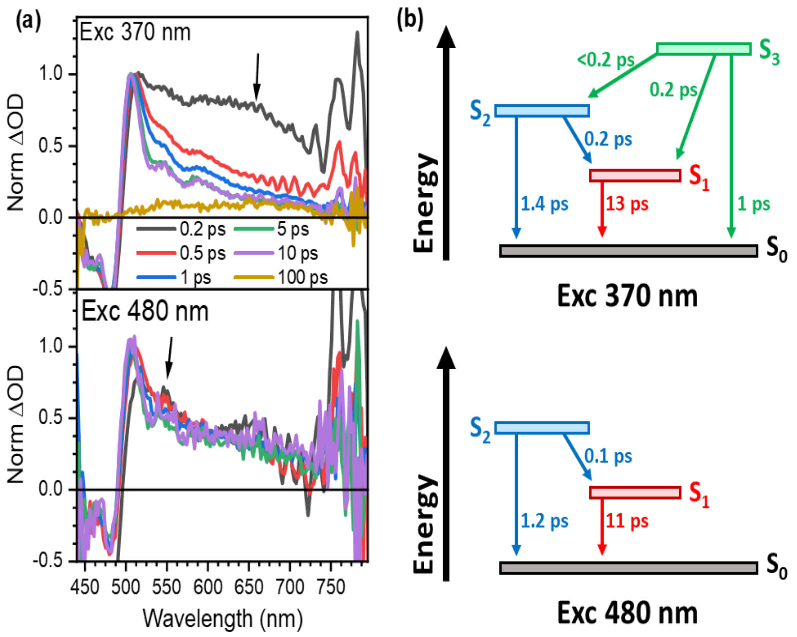

Figure S12. a) Normalised transient absorption spectra of a CH_2Cl_2 solution of **1** recorded at several time delays upon excitation at 370 and 480 nm (top and bottom, respectively) at 0.25 mW. B) Jablonski diagram depicting the processes seen in the TAS experiment.

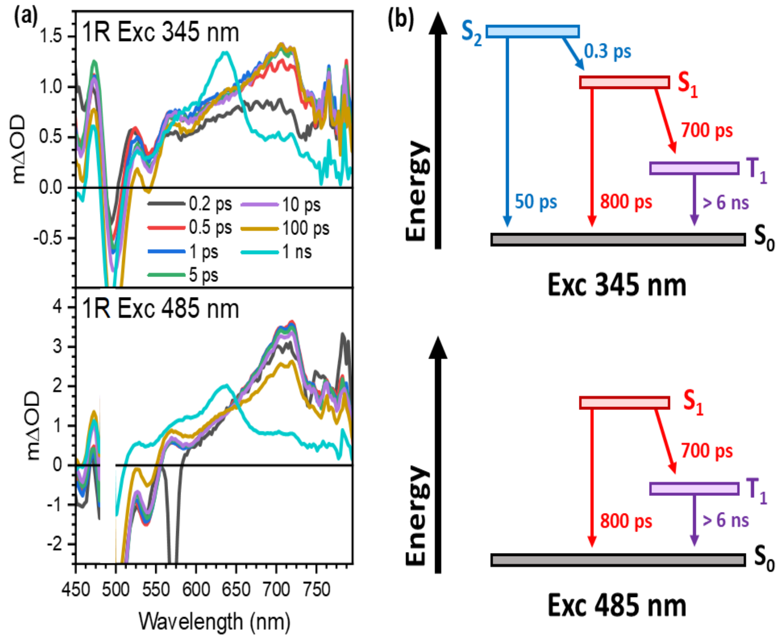

Figure S13. a) Transient absorption spectra of a CH_2Cl_2 solution of **2** recorded at several time delays upon excitation at 345 and 485 nm (top and bottom, respectively) at 0.25 mW. b) Jablonski diagram depicting the processes seen in the TAS

Ground state and time resolved fluorescence characterization has been carried out in an Edinburgh fluorimeter FLS920. With a 400W Xenon lamp Xe900 and a R2658P. For nanosecond time resolved characterization pulsed laser EPL375 and an ultrafast F-G05 detector have been used.

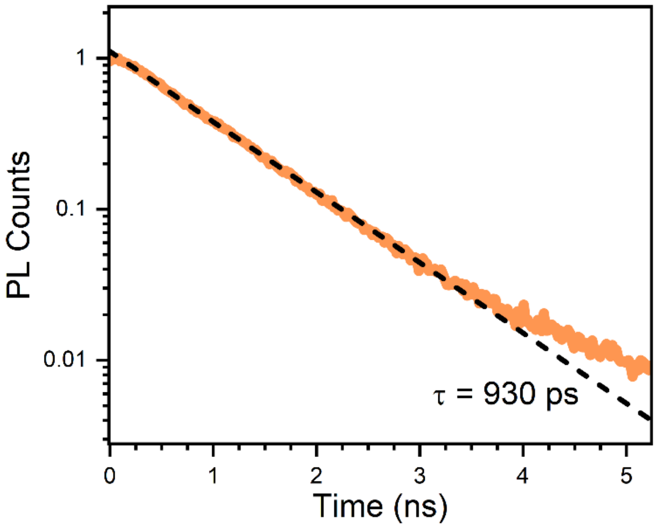

Figure S14. Fluorescence decay of a CH_2Cl_2 solution of **2**. The dark dashed line indicates the monoexponential fitting for the decay with a lifetime of 930 ps.

3. Optimized coordinates

Table S16. Ground state optimized molecular geometry (in Angstroms) of molecule **1** obtained at the CAM-B3LYP/cc-pVDZ level.

C	-5.452832	-0.909878	0.296671
C	-6.465283	-1.555435	-0.419672
H	-6.208088	-2.392724	-1.071314
C	-7.787698	-1.138224	-0.321018
H	-8.544388	-1.667648	-0.902720
C	-8.162333	-0.068824	0.501617
C	-7.144326	0.566149	1.219417
H	-7.376517	1.399356	1.881185
C	-5.817350	0.157451	1.119103
H	-5.049535	0.676208	1.695716
C	-4.044449	-1.391544	0.228551
C	-3.754554	-2.648671	0.690927
H	-4.555916	-3.265052	1.102353
C	-2.434378	-3.181381	0.688802
C	-1.343471	-2.415318	0.176508
C	-1.627394	-1.055953	-0.159289
C	-2.948442	-0.586434	-0.194317
C	-9.634370	0.354321	0.588562
C	-9.842650	1.532265	1.546206
H	-10.909610	1.798685	1.577410
H	-9.287383	2.426064	1.224610
H	-9.531952	1.285648	2.572295
C	-2.208624	-4.474378	1.215504
H	-3.054092	-5.025007	1.632510
C	-0.948442	-5.011729	1.215313
H	-0.758624	-5.991996	1.655258
C	0.100973	-4.311650	0.597086
H	1.085097	-4.776982	0.551849
C	-0.073715	-3.056942	0.029152
C	-0.720142	0.056452	-0.407016
C	1.071089	-2.531952	-0.779330
C	1.488338	-1.149820	-0.684842
C	0.727200	-0.028983	-0.410187
C	1.635600	1.082115	-0.160557
C	1.353197	2.440305	0.181136
C	0.083355	3.083050	0.039712
C	-1.064778	2.560719	-0.765701
C	-1.482378	1.178502	-0.673679
C	-2.905633	0.770587	-0.716536
C	-10.130016	0.773195	-0.806167

H	-11.188492	1.074566	-0.761912
H	-10.045628	-0.048014	-1.532235
H	-9.548034	1.624062	-1.191778
C	-10.476546	-0.830460	1.092449
H	-11.538015	-0.544108	1.160668
H	-10.144510	-1.153738	2.090710
H	-10.406759	-1.696251	0.418186
C	1.709029	-3.478639	-1.550698
H	1.192719	-4.435782	-1.629842
C	2.911501	-0.741593	-0.731315
C	3.952694	-1.333415	-1.387099
H	4.897649	-0.790784	-1.377519
C	3.939330	-2.513550	-2.181832
H	4.844047	-2.704442	-2.763376
C	2.936456	-3.431792	-2.270132
H	3.120317	-4.293191	-2.918194
C	2.956270	0.612491	-0.201945
C	4.054351	1.415213	0.220360
C	3.766508	2.670957	0.687772
H	4.569486	3.285828	1.098388
C	2.446397	3.204154	0.692065
C	-0.088805	4.336343	0.611652
H	-1.072731	4.802479	0.570072
C	2.222747	4.495449	1.223721
H	3.069457	5.043861	1.641056
C	0.962911	5.033646	1.229029
H	0.775133	6.012643	1.672603
C	-1.704720	3.509510	-1.532921
H	-1.186647	4.465611	-1.613195
C	-3.950073	1.366266	-1.363592
H	-4.895085	0.823870	-1.351590
C	-3.940797	2.550110	-2.152561
H	-4.849183	2.745046	-2.727026
C	-2.936880	3.466861	-2.244466
H	-3.123882	4.330968	-2.887898
C	5.461548	0.929880	0.282724
C	6.474679	1.578396	-0.421461
H	6.222462	2.426399	-1.061132
C	7.801052	1.154155	-0.332821
H	8.552410	1.692431	-0.909269
C	8.167905	0.072791	0.469305
C	7.142803	-0.566535	1.182800
H	7.379218	-1.410732	1.832840
C	5.821752	-0.153720	1.093255

H	5.051675	-0.675810	1.663697
C	9.613434	-0.424186	0.595154
C	10.582938	0.391655	-0.266182
H	11.603213	-0.003788	-0.150865
H	10.601926	1.451037	0.029686
H	10.325012	0.337950	-1.334301
C	9.690558	-1.893153	0.144859
H	10.722076	-2.267307	0.239486
H	9.382528	-1.998354	-0.906513
H	9.042279	-2.541484	0.751760
C	10.063987	-0.317210	2.062174
H	11.099480	-0.676662	2.170532
H	9.429391	-0.918403	2.729277
H	10.024335	0.725823	2.411040

Table S17. Molecular geometry (in Angstroms) of the minimum on the S_3 state potential energy surface of molecule **1** optimized at the CAM-B3LYP/cc-pVDZ level.

C	-5.459860	-0.893270	0.228920
C	-6.465000	-1.550550	-0.486950
H	-6.201200	-2.395210	-1.126420
C	-7.789280	-1.135580	-0.403750
H	-8.539960	-1.673750	-0.985380
C	-8.173260	-0.057490	0.402920
C	-7.162290	0.589440	1.120170
H	-7.401630	1.430030	1.769880
C	-5.833730	0.183240	1.035370
H	-5.072060	0.711240	1.611780
C	-4.051060	-1.376430	0.186410
C	-3.777480	-2.635350	0.664790
H	-4.594330	-3.246260	1.053240
C	-2.463420	-3.175490	0.708410
C	-1.347290	-2.413430	0.230740
C	-1.613540	-1.056370	-0.116280
C	-2.943840	-0.582570	-0.214770
C	-9.646490	0.364320	0.470570
C	-9.869370	1.537090	1.430980
H	-10.937680	1.799230	1.453250
H	-9.315200	2.434710	1.118490
H	-9.567120	1.287740	2.459000
C	-2.267160	-4.465210	1.246820
H	-3.129250	-5.007850	1.638790
C	-1.008150	-5.014130	1.284200
H	-0.839980	-5.994210	1.733320

C	0.061070	-4.329620	0.695240
H	1.040420	-4.805940	0.680340
C	-0.078400	-3.063730	0.123190
C	-0.706690	0.051870	-0.306920
C	1.095910	-2.552030	-0.632600
C	1.496660	-1.191840	-0.588110
C	0.712850	-0.033420	-0.306210
C	1.619480	1.074330	-0.112870
C	1.353020	2.430900	0.235750
C	0.084590	3.081770	0.126810
C	-1.088970	2.570920	-0.630480
C	-1.490250	1.210650	-0.587240
C	-2.889620	0.769490	-0.725580
C	-10.119850	0.790400	-0.929610
H	-11.179830	1.088510	-0.901670
H	-10.019870	-0.025720	-1.659720
H	-9.533710	1.645420	-1.299460
C	-10.496860	-0.822380	0.955430
H	-11.558700	-0.534680	1.009980
H	-10.179470	-1.151880	1.956460
H	-10.419620	-1.684720	0.277620
C	1.785700	-3.558050	-1.346630
H	1.248540	-4.503020	-1.416910
C	2.895760	-0.749610	-0.726970
C	3.954040	-1.349700	-1.381920
H	4.866400	-0.757670	-1.440850
C	3.990430	-2.585230	-2.054120
H	4.899980	-2.806630	-2.615650
C	3.005510	-3.552430	-2.033510
H	3.219820	-4.468560	-2.591260
C	2.950080	0.600920	-0.212080
C	4.057140	1.394350	0.190980
C	3.782830	2.652050	0.672490
H	4.599420	3.262350	1.062340
C	2.468780	3.192120	0.715810
C	-0.055230	4.347640	0.698920
H	-1.034290	4.824520	0.682370
C	2.272070	4.481460	1.254970
H	3.133730	5.023490	1.648740
C	1.013300	5.031190	1.290150
H	0.844910	6.011220	1.739290
C	-1.778060	3.577450	-1.344090
H	-1.240150	4.522020	-1.414280
C	-3.948960	1.371800	-1.376490

H	-4.862530	0.781290	-1.432870
C	-3.985160	2.607730	-2.048070
H	-4.895610	2.830530	-2.607610
C	-2.998750	3.573360	-2.029610
H	-3.212590	4.489690	-2.587200
C	5.466500	0.913120	0.226760
C	6.466860	1.578140	-0.480530
H	6.201330	2.432180	-1.106680
C	7.796980	1.161890	-0.413200
H	8.538060	1.711760	-0.991810
C	8.180300	0.072880	0.370230
C	7.168270	-0.582720	1.088010
H	7.418860	-1.434380	1.722980
C	5.843680	-0.177790	1.019930
H	5.083930	-0.712480	1.592650
C	9.630550	-0.415820	0.471330
C	10.583640	0.418410	-0.390620
H	11.607180	0.025570	-0.297470
H	10.602810	1.472840	-0.077370
H	10.310110	0.381260	-1.455560
C	9.711660	-1.877830	-0.000520
H	10.746250	-2.247530	0.077220
H	9.393100	-1.970790	-1.050040
H	9.073690	-2.538640	0.603950
C	10.098050	-0.325370	1.933800
H	11.138020	-0.676460	2.025560
H	9.476890	-0.941190	2.600210
H	10.053430	0.712430	2.297390
